# Anti-CD19 chimeric antigen receptor T-cell followed by interferon−α therapy induces durable complete remission in donor cell-derived acute lymphoblastic leukemia: A case report

**DOI:** 10.3389/fonc.2022.1021786

**Published:** 2022-11-24

**Authors:** Jing Ni, Junjie Zhou, Zhangbiao Long, Xin Chen, Xiaowen Chen, Jian Hong, Xinglin Liang, Qingsheng Li, Ruixiang Xia, Jian Ge

**Affiliations:** ^1^ Department of Hematology, The First Affiliated Hospital of Anhui Medical University, Hefei, Anhui, China; ^2^ Department of Hematology, Xuanwu Hospital, Capital Medical University, Beijing, China

**Keywords:** chimeric antigen receptor T cell, interferon-α, durable complete remission, donor cell-derived leukemia, post-transplant recurrence

## Abstract

Donor cell-derived leukemia (DCL) is a special type of relapse after allogeneic hematopoietic stem cell transplantation (allo-HSCT). Patients with DCL generally have a poor prognosis due to resistance to conventional chemotherapy. Here, we report a case of donor cell-derived acute lymphoblastic leukemia after umbilical cord blood transplantation. The patient didn’t respond to induction chemotherapy. She then received anti-CD19 CAR-T cell therapy and achieved MRD-negative complete remission (CR). However, MRD levels rose from negative to 0.05% at 5 months after CAR-T cell therapy. Higher MRD levels were significantly associated with an increased risk of leukemia recurrence. Afterward, preemptive interferon-α treatment was administrated to prevent disease recurrence. To date, the patient has maintained MRD-negative CR for 41 months. Our results suggested that anti-CD19 CAR-T cells followed by interferon-α therapy are effective in treating donor cell-derived acute lymphoblastic leukemia. This report provides a novel strategy for the treatment of DCL.

## Introduction

Leukemia relapse remains one of the most common causes of posttransplant mortality (1). The relapse clone is usually host-derived. Rarely, acute leukemia can also develop *de novo* in donor-derived cells, which is known as donor cell-derived leukemia (DCL). Since the first case of DCL was published in 1971, reports of DCL have accelerated in recent years (2, 3). Available data suggested that DCL accounts for approximately 5% of posttransplant relapses (4). The prognosis of DCL was poor, with a median survival time of 6 months after diagnosis (5). A second allogeneic hematopoietic stem cell transplantation (allo-HSCT) after successful re-induction remission seems to be an effective means for achieving long-term survival. However, most DCL patients are resistant to chemotherapy and fail to achieve remission again. Furthermore, a second allo-HSCT is also difficult to implement due to the patient’s poor physical function, lack of suitable donors, etc.

CD19-targeted chimeric antigen receptor T-cell (CAR-T cell) therapy is a promising treatment for relapsed/refractory B-cell acute lymphoblastic leukemia (r/r B-ALL) with a high complete remission (CR) rate of 70–90% (6, 7). Nevertheless, the long-term efficacy of CAR-T cell therapy remains unsatisfactory due to the high recurrence rate, with a median event-free survival of only 6.1 months (8). There is a growing need for new strategies to prevent relapse and maintain sustained remission after CAR-T cell therapy.

Interferon-α is a biological agent with anti-leukemic effects (9, 10). Previous studies have demonstrated that interferon-α reduces the relapse of leukemia and improves long-term survival after chemotherapy and allo-HSCT (11, 12). However, whether the use of interferon-a following CAR-T cell therapy can reduce relapse has not been reported in clinical settings. In this paper, we for the first time present a successful case of treating donor cell-derived B-ALL with anti-CD19 CAR-T cell therapy followed by interferon-α. The patient achieved minimal residual disease (MRD) -negative CR after CAR-T cell therapy. However, MRD rose 5 months after CAR-T cell therapy. Subsequent treatment with interferon-α allowed the patient to regain undetectable MRD. To date, the patient has remained CR for 41 months after CAR-T cells followed by interferon-α therapy.

## Case description

**Figure 1:**
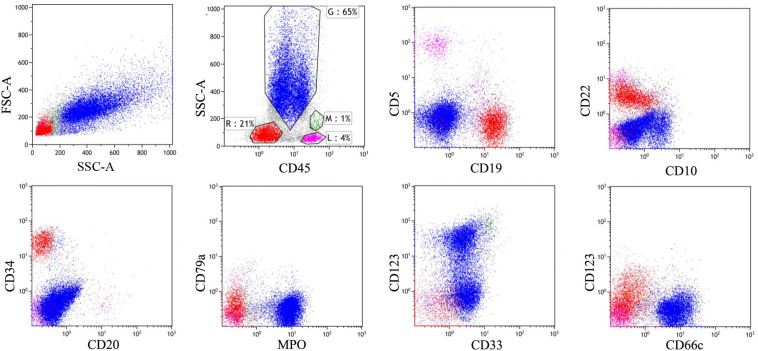
Immunophenotypic analysis by flow cytometry revealed a common B-cell acute lymphoblastic leukemia. There were 21% lymphoblasts expressing CD19+ in the bone marrow at the time of post-transplant recurrence, represented in red.

**Figure 2:**
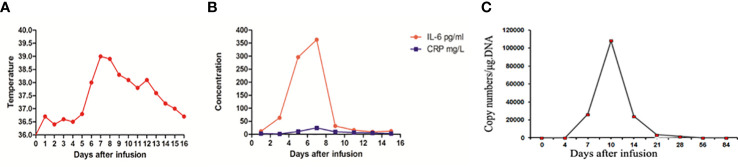
Clinical evolutions after CAR-T cells infusion. **(A)** Measures of body temperature after CAR-T cells infusion, the plot showed the maximum temperature was 39°C on day 7. **(B)** Levels of cytokines IL-6 and CRP were monitored at the indicated time points after CAR-T cells infusion. IL-6 and CRP peaked on day 7. **(C)** The vector copy number of anti-CD19 CAR in peripheral blood was detected by PCR. The highest anti-CD19 CAR DNA copy number was 108,140 copies/µg DNA on day 10. IL-6, interleukin-6; CRP, C reactive protein; PCR, polymerase chain reaction.

**Figure 3:**
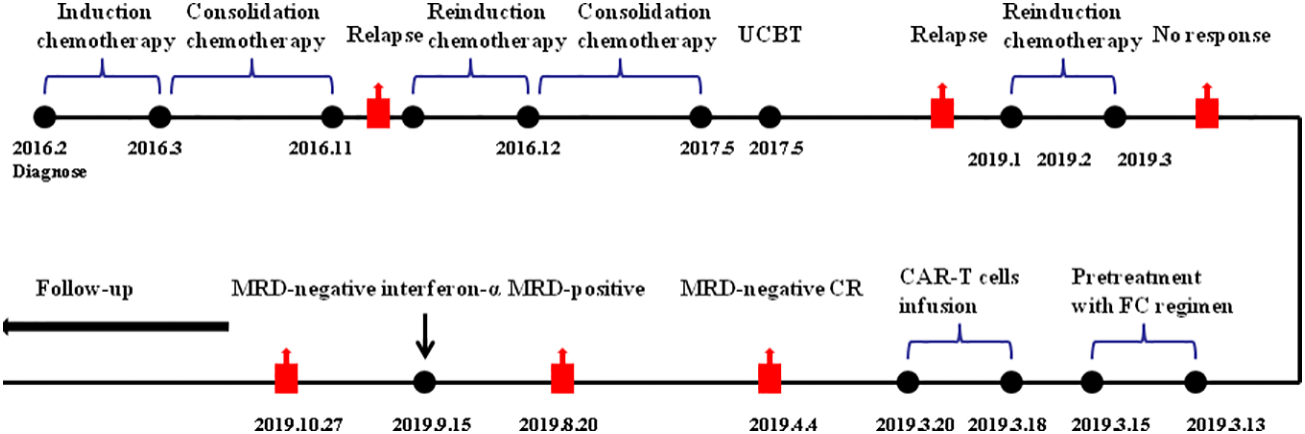
Summary of clinical course. FC, fludarabine and cyclophosphamide regimen; CAR-T cell, chimeric antigen receptor T cell; MRD, minimal residual disease; IFN- α, interferon-α.

An 18-year-old female was admitted to the hospital with abdominal pain on January 31, 2016. Routine blood test revealed white blood cell count of 102.52×10^9^/L, hemoglobin levels of 6.9 g/dL, and platelet count of 57×10^9^/L. Bone marrow smear and flow cytometry identified 90% of blast cells expressing CD34, HLA-DR, CD19, CD10, and CD22. Cytogenetic analysis showed a normal 46, XX karyotype. RT-PCR analysis did not detect any gene rearrangements, such as ETV6, KMT2A, ABL1, ABL2, JAK2, IKZF1, CRLF2, PDGFR*B*, TCF3, ZNF384, and CSFF1R. Therefore, the patient was diagnosed with B-ALL (common-B, poor-risk group). On February 9, she received induction chemotherapy with VIP regimen (vincristine, idarubicin, and dexamethasone) and successfully achieved CR. Next, consolidation chemotherapy including VILP (VIP+ asparaginase) and hyper-CVAD regimen was performed. The patient refused allo-HSCT at the time of the first CR. In November 2016, bone marrow examination showed 41% lymphoblastic cells, indicating leukemia relapse. After re-induction therapy with VILP regimen, the patient attained her second CR. She subsequently underwent an umbilical cord blood transplantation (UCBT) in May 2017. Allografts were from a 4/6 HLA-matched unrelated umbilical cord blood according to low-resolution. The doses of total nucleated cells and CD34+ cells infused were 3.9×107/kg and 3.0×105/kg, respectively. The conditioning regimen included intravenous busulfan at 3.2 mg/kg/d for 4 days, cyclophosphamide at 60 mg/kg daily for 2 days, and fludarabine at 30 mg/m2 daily for 4 days. Cyclosporine and mycophenolate mofetil were used for GVHD prophylaxis. Neutrophil and platelet engraftment was observed on day 22 and day 37 after umbilical cord blood infusion, respectively. The patient maintained CR for 19 months with complete donor chimerism and without graft-versus-host disease. However, in January 2019, 21% of lymphoblasts were detected in the bone marrow by morphological analysis and flow cytometry (**Figure 1**). The patient relapsed again after UCBT. Intriguingly, chimerism testing using short tandem repeats still showed complete donor chimerism at this time point. This was termed “donor cell-derived leukemia”(DCL). Chemotherapy with VIP regimen failed to induce remission (32% lymphoblasts in bone marrow). The patient had no opportunity for second transplantation due to lack of available donors. CAR-T cell therapy was recommended for the patient. She was enrolled in our clinical trial of anti-CD19 CAR-T cell therapy (ChiCTR1800016315). After informed consent, peripheral blood lymphocytes were collected from the patient to prepare anti-CD19 CAR-T cells, which were engineered by Gracell Biotechnologies of Shanghai, China. The patient received lymphodepletion pretreatment with FC regimen (fludarabine 30 mg/m2 daily for 3 days, cyclophosphamide 300 mg/m2 daily for 3 days) from March 13, 2019 to March 15, 2019. After the lymphodepletion pretreatment, anti-CD19 CAR-T cells were infused at a total dose of 2.0×10^6^/kg for 3 consecutive days (0.2×10^6^/kg at day 0, 0.6×10^6^/kg at day 1, 1.2×10^6^/kg at day 2). Body temperature, cytokine levels, and c-reactive protein (CRP) were monitored (**Figures 2A, B**). The patient developed a fever with a maximum body temperature of 38°C on day 6. The temperature rose to 39°C and the blood pressure dropped to 83/58 mmHg on day 7. According to the standard of the American Society for Transplantation and Cellular Therapy (ASTCT), the patient was diagnosed with grade 2 cytokine release syndrome (CRS) (13). Non-steroidal anti-inflammatory drugs (NSAIDs) and tocilizumab (8 mg/kg) were used for CRS. Afterward, the temperature dropped gradually and returned to normal after a week. The highest serum level of interleukin 6 (IL-6) was 363 pg/mL on day 7. The copy number of anti-CD19 CAR in peripheral blood reached its peak on day 10, which was 1.08×10^5^ copies/μg.DNA (**Figure 2C**). On April 4, 2019 (day 17), the bone marrow smear found no lymphoblast, and FCM revealed MRD negative (MRD<0.01% by 8-color flow cytometry). On day 118 after CAR-T cells infusion, the copy number of CAR was not detected. The patient remained in MRD-negative status for 5 months. On August 20, 2019, flow cytometry examination showed MRD levels of 0.05% for 2 consecutive bone marrow samples within a 1-month interval. The immunophenotype of MRD was CD34+CD10+CD19+CD22+ CD58dim CD20-CD123-. Given the increased MRD levels, the patient was given 3 million IU of interferon-α-2b, three times a week. She achieved MRD negative again after 42 days of interferon-alpha treatment. Interferon-α-2b was used for 2 years. Notably, analysis of immune cell subsets from peripheral blood showed a significant increase in the proportion of CD16^+^ CD56^+^ NK cells. To date, the patient has maintained CR for 41 months after CAR-T cells followed by interferon-α therapy. No adverse reactions were observed during interferon-α treatment. The patient is still under follow-up now. The main clinical process of the patient is summarized in **Figure 3**.

## Discussion

Here, we reported a case of donor cell-derived B-ALL treated with anti-CD19 CAR-T cells followed by interferon-α treatment. The patient developed DCL 20 months after UCBT. Leukemia cells were resistant to chemotherapy at this time. She then received anti-CD19 CAR-T cell therapy and achieved MRD-negative CR. However, after 5 months of CAR-T cell therapy, MRD levels rose to 0.05%. Based on the sensitivity of EuroFlow 8-color flow cytometry, MRD ≥0.01% was defined as MRD positive (14). The rising MRD levels indicated an increased rate of leukemia recurrence (15). Fortunately, the patient regained MRD-undetectable after interferon-α treatment. To date, the patient has remained in CR for 41 months. Our case suggested that CAR-T cell therapy followed by interferon-α had excellent clinical efficacy in DCL. To the best of our knowledge, this is the first successful clinical case of DCL treated with CAR-T cell therapy followed by interferon-α.

DCL is a rare and serious complication after allo-HSCT. UCBT was a risk factor for DCL compared to bone marrow transplantation. DCL following UCBT tends to be resistant to chemotherapy and the prognosis is very poor (5). The median interval between the occurrence of DCL following CBT was 14.5 months (16). Consistent with the characteristics reported in previous studies, the patient in our case developed DCL 20 months after CBT and was resistant to chemotherapy. Mechanistically, impaired immune surveillance by reduced functional T-lymphocytes in the recipient microenvironment was considered to promote the occurrence of DCL (16). Thus, restoring donor T cell functions, such as adoptive T cell therapy may be a promising approach for DCL therapy. Adoptive cell therapy using CAR-T cells has demonstrated impressive responses in treating r/r B-ALL. Thus, anti-CD19 CAR-T cell therapy was used to treat donor cell-derived B-ALL in this case. As expected, the patient achieved MRD-negative CR.

Despite the high remission rate, the long-term survival of B-ALL patients after CAR-T cell therapy is still unsatisfactory. Relapse remains a major challenge, especially for patients who are MRD positive after CAR-T cell therapy. Park et al. found that all 9 patients with MRD-positive CR after CAR-T cell therapy experienced relapse, indicating a 100% relapse rate (8). Bridging to allo-HSCT is considered an important means to reduce relapse after CAR-T cell therapy (17). However, a substantial proportion of patients are not eligible for allo-HSCT due to lack of suitable donors, patients’ poor physical function, and so on. Here, MRD level of this patient was significantly elevated after 5 months after CAR-T cell therapy. A second allo-HSCT was not feasible due to lack of suitable donors. Therefore, there is a strong need to adopt novel therapeutic approaches to prevent relapse in this patient.

Interferon-α is a cytokine that can directly inhibit the proliferation of leukemia cells (11). More importantly, it has an immunoregulatory function, which is a crucial mechanism against leukemia. Interferon-α induces the activation and maturation of DCs, enhances cytotoxic activities of natural killer cells, and amplifies the proliferation and activation of T lymphocytes (18). It also significantly increases human CD8^+^T cells exhibiting a surface phenotype of T central memory cells, which helps induce profound and sustained remission in leukemia patients (19). Based on the immunomodulatory effect, interferon-α promotes sustained remission by increasing the number of memory T cells and NK cells in patients with chronic myeloid leukemia (20). Interferon-α also promotes the graft-versus-leukemia effect. Therefore, it is used as an adjuvant or maintenance treatment after allo-HSCT to clear MRD and reduce the recurrence of leukemia (12, 21). Of note, several studies have reported synergistic effects between interferon-α and cell therapy. For example, interferon-α was found to increase the efficacy of donor lymphocyte infusion in posttransplant patients (22). Interferon-α enhanced the killing effects of CAR-T cells *in vitro* by increasing CAR-T cell activation and cytokine production (23). However, the clinical application of CAR-T cells combined with interferon-α treatment has not been reported. In this report, we observed that CAR-T cell followed by interferon-α therapy induced a durable remission in this patient with DCL. The mechanism underlying the efficacy of interferon-α in the case requires exploration. We found that the copy numbers of CAR were not detected when interferon-α was used, indicating that interferon-α did not work by enhancing CAR-T activity. The patient remained in complete donor chimerism, suggesting that interferon-α might induce a graft-versus-leukemia effect through immunomodulation.

In conclusion, the present case suggested that anti-CD19 CAR-T cell followed by interferon-α treatment was effective in donor cell-derived B-ALL. Based on the possible mechanism, we envision that not only for patients with DCL, but also for all leukemia patients who relapse after allo-HSCT, sequential interferon-α therapy helps maintain durable remission if the patient achieves complete remission and donor chimerism after CAR-T cell therapy. Although the results of this report are encouraging, more studies are required to evaluate the efficacy of this treatment strategy.

## Data availability statement

The original contributions presented in the study are included in the article/Supplementary Material. Further inquiries can be directed to the corresponding author.

## Ethics statement

Written informed consent was obtained from the individual for the publication of any potentially identifiable images or data included in this article.

## Author contributions

JN and JZ drafted the manuscript. ZL, XinC, XiaC, JH, and XL took care of the patient. JN,QL and JG collected clinical data. JN and JG analyzed the data. RX and JG revised the manuscript. All authors contributed to the article and approved the submitted version.

## Funding

This work was supported by Key research and development plan of Anhui Province(Grant No. 201904a07020057), Natural Science Foundation of Anhui province (Grant No. 2108085MH270), Research Foundation of Anhui Provincial Institute of Translational Medicine(Grant No. 2021zhyx-C32), Research Foundation of Anhui Medical University (Grant No.2020xkj166 and No.2021xkj154), Clinical Trial Initiative Projects of The first Affiliated Hospital of Anhui Medical University(Grant No. LCYJ2021YB009).

## Conflict of interest

The authors declare that the research was conducted in the absence of any commercial or financial relationships that could be construed as a potential conflict of interest.

## Publisher’s note

All claims expressed in this article are solely those of the authors and do not necessarily represent those of their affiliated organizations, or those of the publisher, the editors and the reviewers. Any product that may be evaluated in this article, or claim that may be made by its manufacturer, is not guaranteed or endorsed by the publisher.
